# Sources of High Variance between Probe Signals in Affymetrix Short Oligonucleotide Microarrays

**DOI:** 10.3390/s140100532

**Published:** 2013-12-31

**Authors:** Roman Jaksik, Michal Marczyk, Joanna Polanska, Joanna Rzeszowska-Wolny

**Affiliations:** 1 Systems Engineering Group, Faculty of Automatic Control, Electronics and Informatics, Silesian University of Technology, Gliwice 44-100, Poland; E-Mail: joanna.rzeszowska@polsl.pl; 2 Data Mining Group, Faculty of Automatic Control, Electronics and Informatics, Silesian University of Technology, Gliwice 44-100, Poland; E-Mails: michal.marczyk@polsl.pl (M.M.); joanna.polanska@polsl.pl (J.P.)

**Keywords:** microarrays, GC-content, custom CDF, G-quadruplex, oligo(dT)

## Abstract

High density oligonucleotide microarrays present a big challenge for statistical data processing methods which aim to separate changes induced by experimental factors from those caused by artifacts and measurement inaccuracies. Despite huge advances in the field of microarray probe design methods, the signal variation between probes that target a single transcript is substantially larger than their between-replicate array variability, suggesting a large influence of various probe-specific effects that introduce bias to the data. In this work we present the influence of probe-related design variations on the expression intensities of individual probes, focusing on five potential sources of high probe signal variance: the GC composition of the probe, the distance between individual probe target sites, G-quadruplex formation in the probe sequence, the occurrence of sequence motifs complementary to the oligo(dT) primer, and the specificity of unrecognized alternative splicing probeset assignment. By focusing on two high quality microarray datasets based on two distinct array designs we show the extent of variance between probes that target a specific transcript providing guidelines for the future design of microarrays and data processing methods.

## 1. Introduction

Oligonucleotide microarrays represent one of the most widely used methods for the characterization of transcript level changes induced by various physical or chemical factors. Despite a wide range of possibilities which allow identification of candidate genes responsible for the observed regulatory events, microarrays require complex statistical methods in order to distinguish changes induced the by experimental factors analyzed, from those which originate from method specificity and measurement inaccuracy. The problem of statistical analysis presents a major challenge and has been addressed in a wide range of publications, but despite the fact that microarrays are used for over ten years many aspects of their design and processes of data analysis remain questionable [1].

The most commonly used 3′ In Vitro Transcription (3′IVT) Affymetrix microarrays consist of probesets usually incorporating 11 Perfect-Match (PM) 25 nucleotide (nt)-long oligonucleotide probes specific to a ∼600 nt region of the transcript's 3′-UTR and an additional set of corresponding mismatch (MM) probes where the central 13th nucleotide is replaced with its complementary equivalent used for the accession of non-specific binding strength. The next generation of Whole Transcript (WT) expression analysis arrays (like the HuGene-1_0-ST) utilize a set of background intensity probes that have no homology to the transcripts of the organism analyzed which are used to estimate the non-specific binding based on the varying GC content of the probes (the number of G and C nucleotides in the sequence). Additionally, the probes are selected based on various gene exons and not only on the 3′-UTRs as in older designs, which allows more precise separation of the intensities for various splice variants. Exon-specific probesets usually comprise four probes, although transcript- or gene-specific sets include on the average over 25 probes (HuGene-1_0-ST), significantly exceeding the probe numbers in older designs. Due to significant differences between both platforms, various approaches to the data analysis are required. Additionally, the platforms vary in the sample preparation procedures knowledge of which is required for the appropriate understanding of the data analyzed.

The basic steps of a microarray experiment include RNA isolation, cDNA synthesis, amplification and labeling, cRNA fragmentation, hybridization, washing and staining and finally a complete surface scan of the microarray. The main differences between WT and 3′ IVT microarrays concern the cDNA synthesis step and result from the need to either achieve a high quality whole transcript amplification or amplification of the 3′-UTR region. 3′IVT microarrays utilize the oligo(dT) primer which, by binding to the 3′UTR region, initiates the cDNA synthesis in the 3′->5′ direction. This approach allows to achieve a very high yield of amplification in the close vicinity of the 3′ region although it is very susceptible to RNA degradation [2]. In contrast, WT microarrays are based on random primers which can attach to various regions of the transcript thereby promoting the cDNA synthesis reaction independently from the 3′ region. Both primers include a T7 polymerase promoter which in the amplification process leads to a significant increase in the amount of target material due to the *in vitro* transcription process. This step produces cRNAs whose sequence is complementary to the isolated RNA molecules. The efficiency of this process has a significant impact on the expression estimates [3] and is believed to be one of the main sources of bias due to the fact that its efficiency depends on the structural properties of transcripts, including their GC composition (GC-rich transcripts are transcribed with lower efficiency) [4] and their ability to form secondary structures [5]. The fragmented cRNA is hybridized with the microarray in a 16 hour-long process which is also very susceptible to variability. Probes which differ in their structural features hybridize to their targets with different dynamics [6], which additionally depend on the reaction conditions (temperature, salt concentration, *etc.*) [7]. Washing and staining steps are used to remove non-specifically bound cRNA and to attach the phycoerythrin-streptavidin complex to the biotinylated C and U nucleotides in the cRNA (3′IVT arrays) or to the terminal nucleotides added by terminal deoxynucleotidyl transferase (TdT). In the scanning process the fluorescence of phycoerythrin, excitated with a laser, is measured by the microarray scanner.

Every step of these experimental procedures is susceptible to factors that can significantly affect the expression estimates, leading to increased between-probe and between-sample variations of non-biological origin. In order to properly interpret the data a comprehensive understanding of these sources of variation is necessary, and despite the large number of potential sources the incorporation of artifacts-aware methods in the standard pre-processing is highly desirable.

Probes appropriately assigned to transcript-specific sets should show a very similar signal with variance affected mainly by the measurement precision of fluorescence level, similar across all samples and probesets in the experiment. In practice the variance of probes from a single probeset is substantially larger [8] and differs significantly between individual probesets, suggesting the influence of various probe specific effects.

The most frequently addressed source of high probe signal variability is inappropriate probeset definition based on inaccurate transcriptomic data [9–11]. Despite this being one of the major problems, since as reported, depending on the platform, the inappropriate definitions can concern over 50% of all probes [9] it's not the only reason for high inter-probe signal variance. In this work we focus on five distinct reasons for high variance of probe signals, other than the well-described problem of inaccurate probeset definitions, using an updated set of chip definition files (CDF's) with probes re-annotated to the most recent version of the RefSeq transcript database [9]. The main goal of this study is to determine the source of high probe signal variance, assessing its influence on the expression estimates and determining the number of probesets affected by a specific factor. Such knowledge might point out not only flaws in the microarray design processes but also provide guidelines for the data processing techniques.

For the purpose of this study we utilize data from two high-quality microarray experiments conducted on two distinct platforms, the MicroArray Quality Control (MAQC) dataset [12] based on a 3′IVT type HG-U133_Plus_2 platform and an Affy set (created by the manufacturer) based on WT type HuGene-1_0-st arrays. These platforms were chosen based on both their high popularity indicated by the large number of samples deposited in the ArrayExpress repository [13] and on the very high number of probes utilized, which should allow conducting a much more comprehensive probe-level study allowing detection of statistically significant differences in the signal intensity related to their structural features.

## 2. Materials and Methods

### 2.1. Microarray Datasets

Affy-HuGene10ST is a Human Gene 1.0 ST Array dataset downloaded from the official Affymetrix website. The dataset includes 33 samples which assess the expression profile of 11 various commercially available tissues, each with three biological replicates. MAQC-HGU133Plus2 is a dataset based on the expression estimates of four samples assessed with HG-U133_Plus_2.0 microarrays, with five replicates for each sample. The experiment was repeated at six different laboratories resulting in a total of 120 microarrays. This dataset was created as a part of the MicroArray Quality Control (MAQC) project [12] (Gene Expression Omnibus ID: GSE5350).

### 2.2. Data Processing Methods

In all analysis steps the raw datasets were analyzed with probes selected based on official Chip Definition Files (CDF) or custom Brainarray ver. 17 RefSeq transcript-based CDFs [9]. Signal intensities of raw probes were extracted using either Bioconductor or our custom application CelExplorer available at www.cellab.polsl.pl. Probe structural features were extracted based on a set of custom scripts implemented in Python (also available on our website). Statistical data analysis (including the creation of all the figures) was conducted in either Bioconductor or Matlab. For signal or signal variance level comparison we used the Mann-Whitney U rank sum test with the significance level set to 0.01.

### 2.3. Intra-Probeset Variance Study

The intra-probeset signal variance was defined as the variance of raw probe signals from a single microarray calculated between probes from a specific set (assigned to a single gene or transcript). In this study we calculated it for each probeset and each microarray independently and presented it as a median of all acquired values. Such statistic was calculated twice based on two distinct probeset definitions (CDF files) described in Section 2.2. For comparison purposes the between-replicate probe variance was used, which is calculated similarly but for individual probes across all technical/biological-replicate microarrays in a given experiment.

The main goal of this study is to assess the variance level of probe signals resulting mainly from differences in their structural properties, comparing to the signal variance which originates from technical of biological differences between microarrays from a specific experiment.

### 2.4. Analysis of Intra-Probeset Variance Sources

In order to determine the influence of each individual features on the variance level of probe signals we examined five probe features that might affect their signal intensity. The main goal of this section is to assess the influence of selected factors on the expression intensity differences between probes originating from technical aspects of the microarray methods used. The following features were considered:

- Probe GC composition—number of G and C nucleotides in the oligonucleotide sequences of the probes
- Probe target location—differences in the location of probe target region in the mRNA sequence, between probes from a single set
- Multiple transcript group assignment—the occurrence of probes within probesets that bind to distinct transcript groups of a single gene
- Spacer-T7 motif—occurrence of CCGCCTCCC motif in the probe sequence which is a part of the oligo(dT) primer
- G-quadruplex formation—the occurrence of (G)4 motif in the probe sequence

All of the features were examined independently using classical statistical methods implemented in Bioconductor.

### 2.5. Feature Importance Study

The main goal of this study it to assess the impact of individual features described in Section 2.3 on the total intra-probeset variance level. The biggest difficulty of this analysis results from the fact that the observed intra-probeset variance originates from the simultaneous influence of several factors which are not easily distinguishable. For this purpose we have chosen the approach proposed in [14], based on the study of predictors importance used to create a decision tree. The tree differentiates probesets based on the variance levels of probe signals (used as attributes) and specific probeset features studied (used as predictors in the tree). The complete tree is analyzed by an algorithm which determines the influence of each variable on its structure, as described in [13]. We assumed that this statistic is proportional to the influence of individual feature on the intra-probeset variance.

This method was chosen since standard statistical tests either focus on individual features only or require them to be discrete (like ANOVA or PVCA [15]), which raises a number of problems on how to properly discretize each individual feature, which has a significant impact on the analysis outcomes. Decision trees are more flexible allowing multiple features to be used in their original continuous scale.

The tree used in this study was created on the basis of five distinct features discussed in this report and the variance levels calculated for individual probe intensities in each separate probeset, based on every single microarray in a given dataset. The corresponding probeset features were defined using individual probe statistics based on the following criteria:

- Probe GC composition—variance of GC composition for probes within the probeset, where the average of GC content in individual set was replaced with the median of GC composition calculated for all probes.
- Probe target location—variance of probe location based on the 25th percentile of distance from the most 3′ probe, calculated using transcript sequences from the Reference Sequence database.
- Multiple transcript group assignment—the variance between probes is expected to be high when there are many probes assigned to various groups of transcript variants (see Results for a detailed explanation). Based on this assumption we defined the following statistic:
  (1) $$Sd=\frac{Km\;\cdot \;Nk}{Ns}$$
  where *Km* is the number of probes in the largest probe group, assigned to a specific set of alternative transcript variants, *NK* is the number of distinct groups of transcript variants targeted by probes in the set, and *Ns* is the number of probes in a probeset.
- Spacer-T7 motif (CCGCCTCCC)—the percentage of probes in a probeset which include this motif with a maximum of one mismatch.
- G-quadruplex formation—the percentage of probes in a probeset which include a (G)_4_ motif.

The construction of decision tree and the assessment of each statistic's influence on its structure were carried out in Matlab according to the methodology described in [14].

## 3. Results and Discussion

### 3.1. Probe Signal Variance

**Figure 1.**
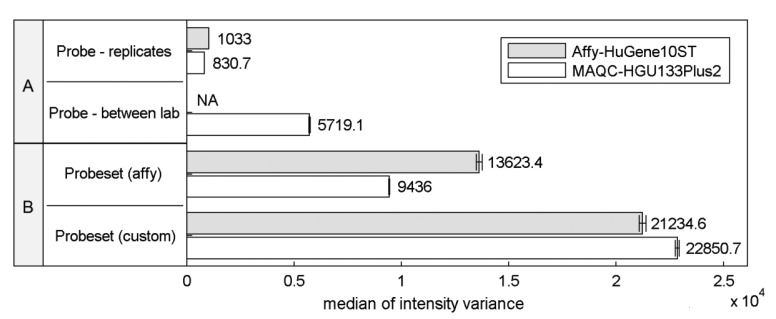
Median of signal intensity variance in two microarray experiments MAQC-HGU133Plus2 and Affy-HuGene10ST with 95% CI. (**A**) median of individual probe intensity variance between biological replicates from a single or various laboratories (**B**) median of probe intensity variance in a single probeset defined using a standard CDF file or a redefined custom CDF based on the up-to-date transcript nucleotide sequence data [9].

Differences in the structural properties between probes mapped to a specific transcript are known to be a reason for high signal variations that can significantly affect the expression estimates. This so called probe-effect substantially exceeds the variation caused by measurement accuracy and between sample differences resulting from batch-effects. Cheng Li showed that the variation across arrays is substantially smaller than the variation between probes in a probeset when considering PM-MM differences [8]. Figure 1 extends this observation to the two commonly-used microarray platforms HuGene-10ST and HG-U133_Plus_2, showing that when considering raw intensity measures the between-array variance of a single probe is significantly smaller than the variance of probes within probesets both as defined by the manufacturer or as customarily redefined based on current genomic databases [9].

This effect is similar even across two distinct platforms, the HG-U133_Plus_2 array with 3′-UTR-specific probes and the exon-based HuGene-10ST. The intra-probeset variance is higher when comparing both variance between technical replicates originating from a single laboratory and between-experiment variation of corresponding samples performed in various laboratories where technical replicates from a single site were averaged reducing the influence of random noise. Between-site samples variance is expected to be higher than the variance of samples conducted in the same laboratory because of experiment-originating batch-effect [16]. Between-laboratory probe signal variance was not calculated for Affy-HuGene10ST experiment since such samples were not provided in this dataset.

Custom probeset assignment aims to increase the signal consistency mainly by excluding probes which do not hybridize to a specific transcript or can hybridize with transcripts of distinct genes. One might expect that this effect should decrease the intra-probeset variance, although in both experiments we observe the opposite behavior. This may result from the omission of specific probeset design criteria, set by the manufacturer, which can lead to an increase of intra-probeset signal variance. One of these criteria is the probe proximity in the 3′IVT microarrays (like HG-U133_Plus_2) which states that all probes should map to a region of at most 600 bp. The Brainarray CDF files [9] which were used in this study do not follow this rule, and therefore suffer from signal differences caused by large probe distances, resulting for example from RNA degradation. The same reason may explain the intra-probeset variance differences between the two platforms observed for the official Affymetrix CDF file. HuGene10ST microarrays include probes located in various exons and therefore spread over large distances exceeding 600 bp, which is characteristic for the 3′IVT designs. In the subsequent sections of this paper we focus on specific factors that may contribute to high intra-probeset variance and additionally address the reasons for the increase of variance in custom CDF-based studies.

### 3.2. Reasons for High Intra-Probeset Variance

#### 3.2.1. Nucleotide Composition of Probes

Difference in oligonucleotide binding strength caused by variations in the GC content is a well-known problem, especially in PCR-based experiments. The percentage of G and C nucleotides determines the melting temperature of the oligonucleotide probe which is defined as the temperature at which half of the hydrogen bonds between the probe and target cDNA break. The melting temperature of Affymetrix probes varies between 40 and 80 °C, and as a consequence of Tm-unaware probeset assignment the average temperature of the entire probeset also shows large variations depending on the platform and CDF file used. If the melting temperature of the probe is higher than the temperature at which hybridization takes place the bonds are stronger, enhancing the process of probe-cDNA binding and thereby enhancing the probe signal due to possible non-specific binding. In turn, a low Tm results in weaker affinities and the overall intensity of low Tm probes is substantially smaller.

**Figure 2.**
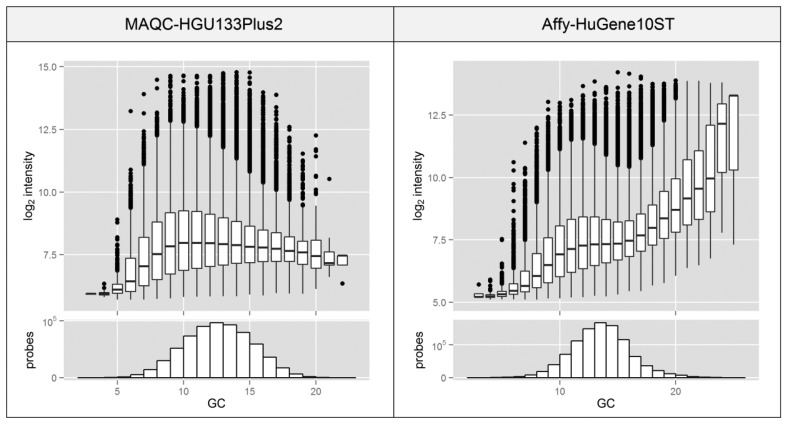
Boxplots representing unprocessed signal intensity of probes and histograms of probe amounts with different number of GC nucleotides in their sequence. Black dots represent outlier probes. Plots are based on data from MAQC and Affy experiments.

Figure 2 shows the relationship between probe GC content (represented as the number of G and C nucleotides in the 25nt-long probe sequence which is proportional to the Tm) and the raw expression intensity. The shape of the plot varies significantly between both analyzed platforms although our experience with other datasets and platforms indicates that it is more experiment-specific than platform-specific. Significant differences in the median intensity levels of probes differing in GC content indicate that GC content can be a major factor that can lead to high intra-probeset variance. The intensity differences are extremely high, especially in the Affy-HuGene10ST experiment where the median intensity of GC-poor and -rich probes differs more than 100-fold on a linear scale. The differences might be much stronger when the specific positions of GC within the probe are considered [17].

The shape of the plot, including the clear decrease of intensity in the region of 15 GC bases, probably reflects a combination of various effects that are GC-content dependent and which have a varying impact on the changes in the intensity distribution. Most of the steps in the experimental procedure can be GC-content dependent especially since the GC content of probes reflects the GC content of transcripts and factors that are affected by the transcript GC levels might also be of importance. The key process that affects the intra-probeset signal variation is probably the hybridization, where high GC probes form stronger bonds which are more resistant to the washing process and are more susceptible to non-specific hybridization, which can artificially increase the signal intensity of GC-rich probes.

#### 3.2.2. G-Quadruplex Formation

The occurrence of a (G)_4_ motif in the probe sequence has been recognized many times in the literature as having a significant impact on the probeset signal integrity [18–22]. Four continuous runs of guanine are believed to form a structure termed a G-quadruplex, making the probe less accessible for the specific target cDNA and sometimes promoting non-specific binding. This is believed to lead to a decreased signal correlation between normal and (G)_4_ containing probes.

**Figure 3.**
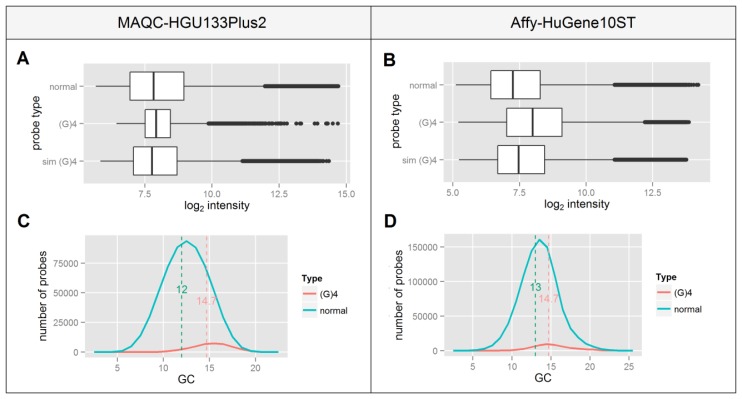
Statistics of probes that contain (G)_4_ motif for both MAQC and Affy datasets (**A**,**B**) signal intensity boxplots of normal probes, probes that contain a (G)_4_ motif and a subset of “normal” probes that have the same GC content distribution as the (G)_4_ probes—“sim (G)_4_” (**C**,**D**) GC content distribution of normal and (G)_4_ probes with medians of both distributions marked with a dashed line.

Since this motif consists only of guanines one can expect that it will occur more frequently in GC-rich probes which is confirmed by panels C and D of Figure 3. This indicates that probes containing a (G)_4_ motif represent a different group of high GC-content probes which as shown in Figure 2, differ in the median signal intensity in an Affy-HuGene10ST experiment. This is consistent with the differences in median signal intensities of normal *vs.* (G)_4_ containing probes shown in panels A and B of Figure 3, which are higher in the Affy-HuGene10ST experiment.

In order to test whether the increase of signal intensity of (G)_4_ probes results solely from the differences in their GC composition or also has a possible background in G-quadruplex formation, we randomly picked a subset of non-(G)_4_ probes in such a way that the distribution of GC content matched ideally to that of the (G)_4_ probes. This process was repeated 1,000 times for each GC value, the resulting data were sorted, averaged and plotted in Figure 3A,B as the “sim (G)_4_” boxplot. In both experiments the difference between the selected probes and the (G)_4_ containing probes is statistically significant (p < 10^−16^) despite their identical GC content distribution. This indicates that the signal differences of (G)_4_ probes might result from factors other than those related to their GC composition, including the formation of G-quadruplexes.

#### 3.2.3. Spacer-T7

Spacer-T7 is the nucleotide motif CCGCCTCCC which is complementary to a fragment of the oligo(dT) primer used to synthesize cDNA in 3′IVT microarray experiments. The sequence complementary to spacer-T7 separates the (T)_24_ sequence (used to attach the starter to the poly-A tail of the transcript) from the T7 RNA polymerase promoter which is crucial for the transcription process used to amplify the material prior to its hybridization with a microarray. Since this fragment is located at the 3′ end of the promoter, it is also transcribed and incorporated into every single cRNA. It has been shown that probes which contain such a motif show significantly higher fluorescence intensity due to non-specific binding with the amplification products [23], despite the fact that it is only 9nt long which is less than half of the entire probe length (25 nt).

Probes which contain the spacer-T7 motif are very rare or non-existent in most of the Affymetrix microarray platforms. The HG-U133_Plus_2 platform (MAQC experiment) has only one such probe while the HuGene-1_0-st (Affy dataset) has merely 69 out of ∼556 thousand. The fact that the signal can be elevated by only partial probe complementarity, which results from nonspecific binding, raises the question of whether the existence of motifs with additional mismatches can cause the same effect.

**Figure 4.**
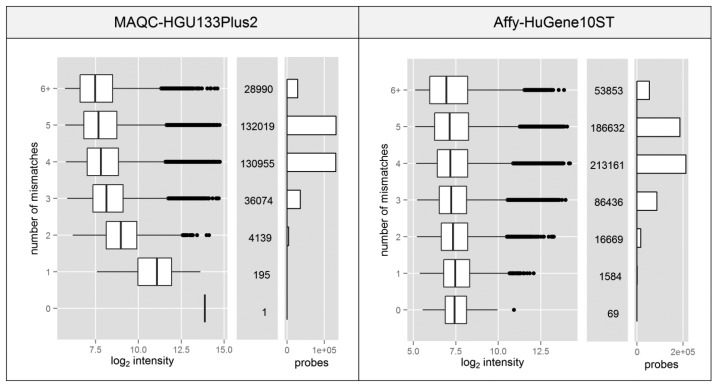
Boxplots of probe signal intensity depending on the occurrence of the T7 motif with zero to over six mismatches. Bar plots and corresponding numbers on the right side of each graph show the number of unique probes used in the selected CDF file for each platform.

Figure 4 shows that even partial probe complementarity to the T7 motif can cause significant elevation of the signal intensity. This only applies to the data from MAQC experiment, since the standard protocol for HuGene10ST microarrays incorporates random primers in place of the oligo(dT).

#### 3.2.4. Probe Target Location

Large distances between probes targeting a single transcript are one of the best-known factors responsible for intra-probeset signal variance. The main reason is degradation of RNA which significantly affects the cDNA synthesis, depending on the method used. 3′IVT microarrays which utilize the oligo(dT) primer suffer from 3′ bias which results in elevated signal intensities of probes located close to the 3′ end of the mRNA [2]. This is caused by the oligo(dT) specificity which causes it to bind to the 3′end of the mRNA triggering the reverse transcription reaction in the 3′->5′ direction. If the transcription fails before reaching the 5′ end, the resulting product will be truncated although it will be used in subsequent steps leading to significant intensity differences between probes which are distant from each other.

**Figure 5.**
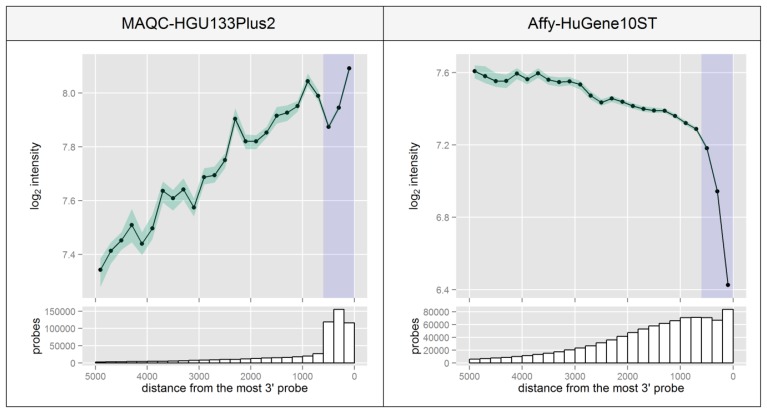
Median and 95% CI of probe intensities relative to the distance of the most 3′ probe in a probeset, calculated for probes in 200nt-long sequence regions. Blue color indicates the 600 bp region defined by the manufacturer as the maximum distance between probes in a single probeset for 3′IVT type microarrays like the HG-U133_Plus_2. Bar plots in the lower panel represent the number of probes which are located at a specific distance from the most 3′ probe.

This phenomenon is used to assess the RNA degradation level as one of the standard steps in microarray quality control, either by comparing the 3′/5′ signal ratios of housekeeping genes (mostly ACTB and GAPDH) or by plotting so-called RNA degradation plots. RNA degradation plots represent the average intensity of probes across all probe-sets, ordered from the 5′ to the 3′ end without knowledge of the precise distances. Due to the specificity of the calculation method this approach can only be applied to 3′IVT arrays in an analysis that utilizes original probeset definitions with a constant probeset size. Our approach presented in Figure 5 aims at overcoming this limitation.

Figure 5 was created based on the precise location of probe targets in the Reference Sequence transcripts, relative to the probe which is located closest to the 3′ end of each transcript. The distances were then divided into 200 bp regions for which a median intensity level was calculated with its 95% CI. In the MAQC experiment the median intensity differences between probes at various distances are very high even in the 600 bp region. The decreasing intensity trend towards the 5′end can be observed also for other distances, although the number of probes that reach such high distances drops significantly in this design which was based solely on the transcript's 3′-UTR. The trend is opposite for the Affy-HuGene10ST experiment since this platform is based on random primers which can attach to various transcript regions, in contrast to the 3′-only oligo(dT). Since the primers can attach to various locations again promoting the reaction in a 3′->5′ direction (even in transcripts with a degraded 3′end), the bias is now towards the 5′ end as shown in Figure 5.

#### 3.2.5. Multiple Transcript Group Assignment

Re-annotation of probes into transcript-specific probesets was shown to increase expression consistency in the entire dataset [24]. It also allows to avoid a number of data analysis issues like multiple probesets assigned to a single transcript [25] or problems with expression intensity assignment to specific sequence-related features of transcripts, like miRNA or protein-binding sites. However, this kind of operation has its drawbacks, and as shown in Figure 1 can lead to an increase in intra-probeset variance. One of the possible reasons may be that a probeset includes probes which not only map to multiple transcript variants (which is very common) but also a distinct set of transcripts expressed at different levels. For example, the CBS gene has three alternative transcript variants NM_001178009, NM_001178008 and NM_000071 and the HG-U133_Plus_2 microarray includes 22 probes specific to that gene, but 16 of these map to all three forms while the remaining 6 target only the NM_001178008 and NM_000071 variants. This may present a potential source of intra-probeset variance if the remaining variant NM_001178009 is expressed at a level different from the other two variants.

**Figure 6.**
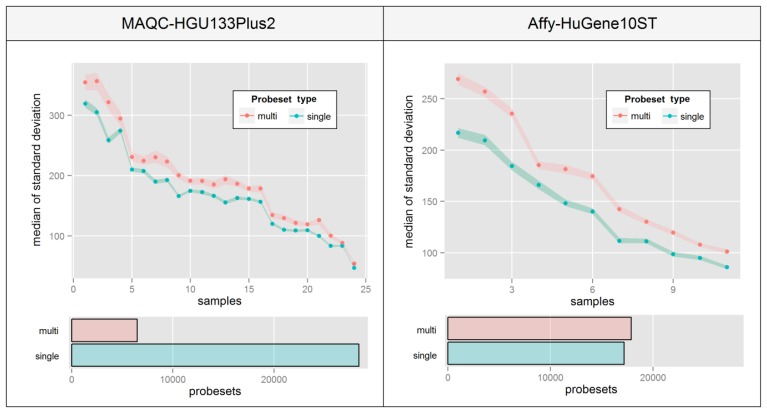
Median standard deviation of intra-probeset intensity levels for probesets that include probes which map to a single or multiple distinct transcript groups (see text for detailed explanation). The figure is created for individual samples in each study comprising of data from three and five averaged microarrays for MAQC and Affy experiment respectively. Bar plots below represent the numbers of both probeset types in each design.

This phenomenon is very hard to visualize since there are no consistencies in the way that probe intensities are affected. Figure 6 aims at overcoming this problem by presenting a median of intra-probeset variance, instead of raw expression estimates, separately for sets that include probes mapping single or multiple distinct transcript groups. The Figure was created by averaging biological replicates and calculating the variance levels separately for individual samples ordered by the average variance level of all probesets. High differences between relative variance levels of individual samples result mainly from raw intensity variations which are typical for un-normalized microarrays.

Figure 6 indicates that in both experiments, probesets which are assigned to multiple transcript groups show much higher intra-probeset signal variance. The differences are much higher in the Affy-HuGene10ST experiment due to the much larger scale of the problem resulting from exon-based probe assignment, which is also the reason for a much higher number of probesets with multi-transcript group assignment. It is worth noting that this effect can also concern gene-specific probeset assignment methods.

The best solution to this problem in the 3′IVT design would be to select probes that are specific to one splice variant, which is in many cases impossible since such probes either do not exist for a given transcript or are in a very low number. This approach would also imply neglecting a very large number of probes from each platform. In such situations approaches based on statistical models like the Gaussian mixture presented in [26] might provide a more comprehensive description of changes in expression levels of various splice variants.

### 3.3. The Impact of Probe Structure Features on the Signal Variance

All of the features analyzed might contribute to the intra-probeset signal variance depending on the specificity of the experimental procedure and the microarray platform used. The main goal of the analysis presented in this section is to determine which features have the highest impact on the variance level and decrease the precision of expression estimates and which have a minor impact on the entire dataset affecting the expression estimates of individual probesets only.

The attempt we have chosen for this task was proposed in [14], where it was applied to a similar microarray problem based on the NimbleGen platform. The method utilizes a decision tree in order to divide the data based on various features that can be represented on a continuous scale. Features of the individual probesets are used as predictors in the tree (see Materials and Methods for their definitions) while the intra-probeset variance levels serve as attributes. The complete tree is analyzed by an algorithm which determines the influence of each variable on its structure. We assumed that this statistic is proportional to the influence of individual feature on the intra-probeset variance.

**Figure 7.**
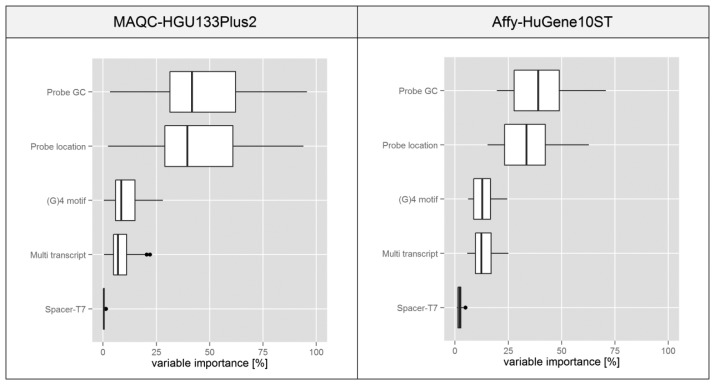
Boxplots representing the influence of individual features on the structure of a decision tree, which we assume reflects their influence on the intra-probeset signal variance.

The boxplots presented in Figure 7 are based on statistics obtained for individual microarrays in a given experiment transformed into a percentage scale based on the median of variable importance score obtained for each feature. In both experiments the plots suggest that variations in the probe GC composition and its binding site location within the transcript have the highest influence on the intra-probeset signal variance. Spacer-T7 has a negligible effect on the global scale since probes containing this motif are very rare, but they might have a very strong influence on individual probesets since as shown in Figure 4 the intensity deviation of those probes can be very strong.

## 4. Conclusions

Our study shows that differences in the nucleotide composition of probes and high distances between their target sites in a transcript sequence are the main reasons for very high intra-probeset signal variance. Additionally, custom chip definition files used in Affymetrix microarray data analysis can lead to a significant increase in the between-probe signal variance and possibly reduce the precision of expression estimates, despite the fact that the number of inappropriately assigned or non-specific probes is significantly reduced in the updated probeset definitions.

It is also worth noting that the estimated contribution of selected factors to the variance level differs significantly between the samples in a given experiment. This raises additional questions as to what is the exact extent to which individual samples from the same dataset are affected, what are the biological/technical sources of those differences and whether they can significantly affect the data analysis process especially the identification of differentially expressed genes. Bias related to the introduced factors, especially GC composition, might lead to false conclusions in microarray based experiments since many of regulatory processes vary between GC-content rich and poor genes [27]. Further understanding of factors that can introduce bias into microarray experiments might contribute to a better understanding of differences in expression estimates between samples, improving the methods of data analysis in both new studies as well as in those conducted many years ago, which results are gathered in publicly available data repositories.
